# The connection between diabetes and depression. Is coaching the best therapeutic path?

**DOI:** 10.1192/j.eurpsy.2022.1928

**Published:** 2022-09-01

**Authors:** I. Farmakopoulou, M. Theodoratou

**Affiliations:** 1 University of Patras, Humanistic Sciences, Patras, Greece; 2 Hellenic Open University, Human Sciences, PAtras, Greece; 3 Neapolis University of Pafos, Health Sciences, Patras, Greece; 4 Hellenic Open University, Human Sciences, Patras, Greece; 5 Neapolis University of Pafos, Health Sciences, Pafos, Cyprus

**Keywords:** diabetes, coping, Depression, coaching

## Abstract

**Introduction:**

As it is well known, diabetes and depression are highly prevalent conditions and affect significantly overall health (Egede & Ellis,2010). This presentation aims to describe the impact of a chronic disease (diabetes) on mental health and its comorbidity with depression. Additionally, it presents the psychotherapeutic process and beneficial effects of coaching on an adolescent patient.

**Objectives:**

The current study tries to present the comorbidity and interaction between diabetes and depression. It explores the therapeutic path followed, so that the patient could cope effectively with the comorbidity of these diseases and break the vicious circle of sadness that he had been into.

**Methods:**

A case study of an adolescent with diabetes is presented. It depicts how the sudden appearance and diagnosis of diabetes led to depressive impasse and cancellation of his dream to become a pilot. The role of coaching is described.

**Results:**

Through monthly coaching the young adolescent discovered his hidden talents and thus he was able to redirect his professional goals and to design a pathway that would lead to the fulfillment of his new life plans. Working closely together with his therapist, step by step, his depressive symptoms were diminished and anti-depressive medication was reduced gradually. Consequently, his diabetes was well regulated, and his overall health was radically improved.

**Conclusions:**

Psychotherapeutic coaching is proved to be appropriate for patients with comorbidity in order to cope effectively with their chronic disease and discover new meaning in their own lives. Last, but not least, self-management skills and diabetes education are required in addition to psychological interventions.

**Disclosure:**

No significant relationships.

